# Aberrant Splicing as a Mechanism for Resistance to Cancer Therapies

**DOI:** 10.3390/cancers17081381

**Published:** 2025-04-21

**Authors:** Duygu Duzgun, Sebastian Oltean

**Affiliations:** 1 University of Exeter, Exeter EX2 4TH, UK; dd388@exeter.ac.uk; 2 Department of Clinical and Biomedical Sciences, Faculty of Health Sciences, University of Exeter, Exeter EX1 2LU, UK

**Keywords:** cancers, chemotherapy, chemoresistance, pre-mRNA alternative splicing

## Abstract

Cancer is biologically diverse, highly heterogeneous, and associated with molecular alterations, significantly contributing to mortality worldwide. Currently, cancer patients are subjected to single or combination treatments comprising chemotherapy, surgery, immunotherapy, radiation therapy, and targeted therapy. Chemotherapy remains the first line of treatment in cancer but faces a major obstacle in the form of chemoresistance. This obstacle has resulted in relapses and poor patient survival due to decreased treatment efficacy. Aberrant pre-mRNA alternative splicing can significantly modulate gene expression and function involved in the resistance mechanisms, potentially shaping the intricate landscape of tumour chemoresistance. Thus, novel strategies targeting abnormal pre-mRNA alternative splicing and understanding the molecular mechanisms of chemotherapy resistance could aid in overcoming the chemotherapeutic challenges. This review first highlights drug targets, drug pumps, detoxification mechanisms, DNA damage response, and evasion of apoptosis and cell death as key molecular mechanisms involved in chemotherapy resistance. Furthermore, the review discusses the progress of research on the dysregulation of alternative splicing and molecular targets involved in chemotherapy resistance in major cancer types.

## Simple Summary

Cancer has consistently posed a significant challenge on a global scale, with rising incidence and mortality. Despite the tremendous advancements in cancer therapeutics throughout the years, chemotherapy remains the current standard treatment in cancer management. As a result, chemotherapy resistance among cancer patients has emerged as a major clinical challenge, causing unsatisfactory responses to the treatment. Chemotherapy resistance is closely related to the dysregulation of a normal cellular process called ‘pre-mRNA Alternative Splicing’, where one gene can generate various protein forms with different structures and functions. A comprehensive understanding of dysregulated alternative splicing mechanisms is essential to propose novel strategies in cancer research aimed at identifying new and improved methods to maximise treatment efficacy and overcome chemotherapy resistance. Therefore, this review highlights the complexity of chemotherapy resistance and the potential roles of alternative splicing in overcoming resistance to chemotherapy.

## 1. Introduction

Cancer is biologically diverse, highly heterogeneous, and associated with molecular alterations, contributing significantly to mortality worldwide [1,2,3]. According to the World Health Organisation Global Reports, 2,001,140 new cancer cases and 611,720 cancer-associated mortalities were predicted globally in 2024 [1]. Given the increasing number of cases diagnosed annually, cancer remains a significant burden on global health [4]. As human life expectancy has increased considerably over the past decade, most cancers have been diagnosed in individuals aged ≥ 65 [5]. This population is expected to grow in the future [6]; thus, researchers continue to seek novel strategies to treat various cancers [7,8,9].

The world has witnessed remarkable advances in recent decades to better understand cancer development, offering promising breakthroughs in cancer treatments [10,11]. Nevertheless, cancer remains an aggressive disease to cure due to several treatment-associated drawbacks [12]. Currently, cancer patients are subjected to single or combination treatments, comprising chemotherapy, surgery, immunotherapy, radiation therapy, and targeted therapy [13,14,15] (Table 1). Chemotherapy remains the first line of treatment in cancer management in combination with surgery and radiation therapy. However, chemoresistance poses a major obstacle in maximising treatment efficacy, resulting in relapses and poor patient survival [9].

**Figure 1:**
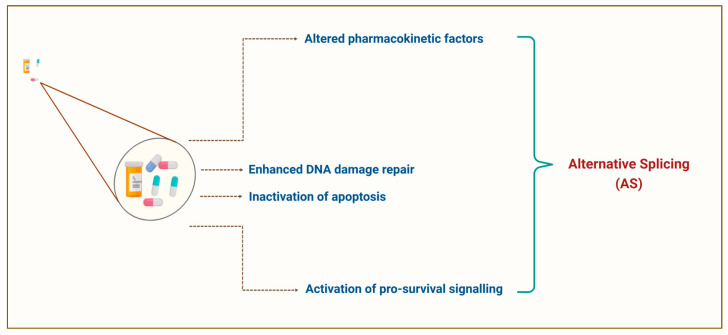
Chemotherapy resistance mechanisms in cancer cells. Created with BioRender.com.

Tumour cells undergo genetic and epigenetic alterations as an escape mechanism, causing cell subpopulations to be unresponsive to chemotherapy and preventing satisfactory responses among cancer patients [20,21]. A subset of these cells are highly adaptable resistant cells, which significantly contribute to chemoresistance development [22,23]. Statistically, 80–90% of cancer mortality is a direct or indirect result of chemoresistance [24,25]. As such, recognising molecular mechanism alterations triggered by chemotherapeutic agents could offer solutions to challenges associated with chemoresistance. These alterations include changes in pharmacokinetic factors, triggering of pro-survival signalling, inactivation of pro-apoptotic signalling, and improved DNA damage repair (see Figure 1) [26,27], arising from activated oncogenic signalling, inhibited tumour suppressor functions, modified drug targets, and changes in metabolism and drug transport. As a result, cancer patients respond poorly to chemotherapy due to the activation of dysregulated apoptotic pathways [20,21].

Numerous genes involved in these processes are regulated by pre-mRNA alternative splicing (AS) (see Figure 1), where specific exons in the mature mRNA are included or excluded [28] to generate different transcript variants of a single gene [29]. More than 95% of human genes are predicted to undergo AS, altering their role, and are occasionally contradictory to their original functions [30,31]. Specifically, aberrant AS and changes in splicing factor abundance promote malignant progression and chemoresistance in tumours by regulating gene expression. Researchers have identified these genes as chemotherapeutic targets to improve treatment efficacy [32,33].

In this review, different mechanisms involved in chemotherapy resistance are summarised, and approaches to improve anti-cancer efficacy via targeting specific mechanisms are discussed. In addition, this review explored how cancer cells benefit from aberrant AS to promote chemoresistance and elude the actions of cytotoxic agents.

This review examines aberrant splicing as a hallmark of chemotherapy resistance, providing a comprehensive overview of current knowledge, highlighting emerging research areas, and delineating strategies targeting splicing dysregulation that potentially overcome chemoresistance.

## 2. The Dark Side of Chemotherapy: Resistance Limits the Success of Chemotherapeutic Agents in Cancer

**Figure 2:**
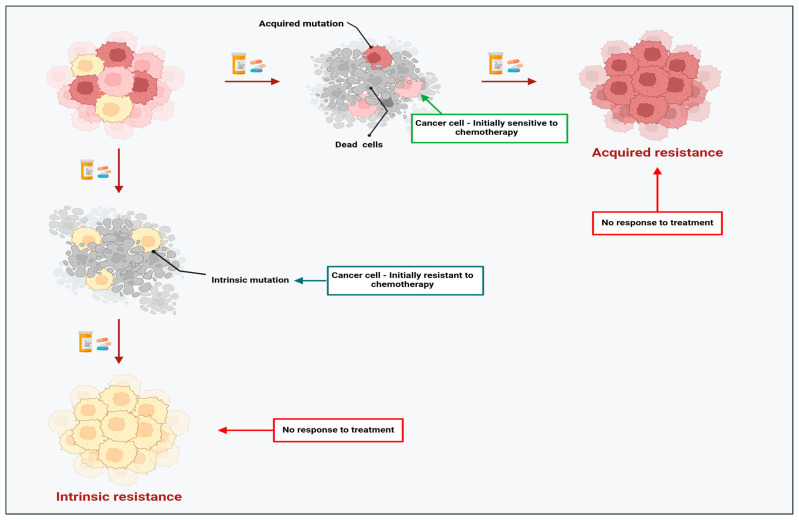
Summary of intrinsic and acquired chemoresistance in cancer cells. Created with BioRender.com.

Despite the advances in cancer therapeutics and the development of newer anti-cancer chemotherapy agents, the development of resistance by tumour cells hampers the success of these treatments [34,35]. It has been reported that over 90% of cancer deaths are linked to chemoresistance [36]. Studies have shown that chemoresistance can be intrinsic (pre-existing) or acquired based on the onset time, which influences disease progression and lethality (see Figure 2) [37,38]. Intrinsic resistance occurs when a subpopulation of heterogenous cancer cells develops resistance naturally in the absence of chemotherapeutic agents; thus, the initial response to treatment is rendered ineffective [39]. In contrast, acquired resistance occurs during cancer treatment, where tumour cells that are initially sensitive to chemotherapeutic agents become unresponsive due to the gradual reduction in the anti-cancer properties of the chemotherapeutic agents. This event leads to intra-tumoral adaptive-resistant subpopulations [40]. Understanding the underlying mechanisms of chemoresistance could reveal how chemotherapeutic drugs effectively trigger cell death in resistant tumour cells.

**Figure 3:**
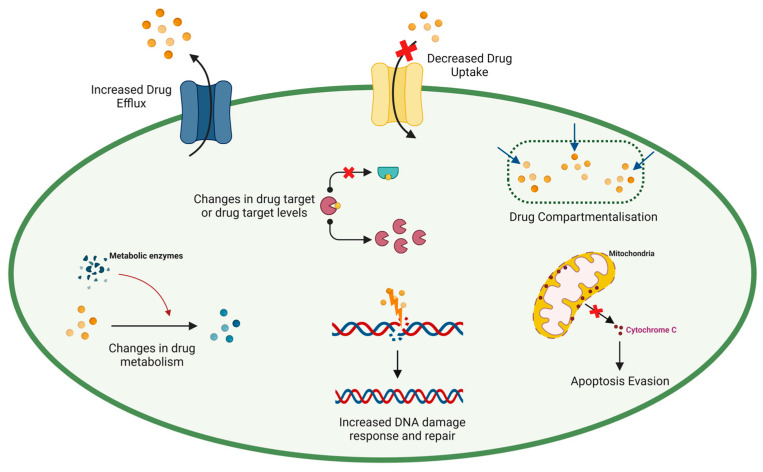
Schematic representation of the five hallmarks that potentially influence chemotherapy resistance mechanisms in cancer cells. Adapted from [24]. Created with BioRender.com.

Five hallmarks have been posited to elucidate the underpinning mechanisms of chemoresistance: inhibition of cell death, detoxification mechanisms, enhanced DNA damage repair, modification of drug targets, and drug pump alteration (see Figure 3) [41,42]. The following subsections discuss these hallmarks in detail.

Alteration of drug targets: The efficacy of chemotherapeutic agents can be restricted by mutations and the abnormal expression of drug targets, which encourages chemoresistance [43]. A meta-analysis report suggested that a mutated tumour suppressor gene p53 led to patients developing treatment resistance against cisplatin, particularly in non-small cell lung cancer (NSCLC) [44]. As a result, Nfr-2 expression is upregulated, causing dysfunction and aberrant pro-apoptotic balance in NSCLC cells [45]. Furthermore, clinical investigations reported that 20–30% of individuals diagnosed with chronic myelogenous leukaemia (CML) developed resistance to imatinib [46,47]. Soverini et al. (2020) revealed that a T315I gatekeeper mutation has been observed in CML patients, preventing imatinib from binding effectively to its target for cytotoxicity, leading to acquired resistance [48].

Alteration of drug pumps: Changes in drug pump configuration can limit and prevent the access of chemotherapeutic agents to targeted tumour cells, thus promoting the development of drug resistance in tumour cells [49]. A significant example of this hallmark is the upregulation of transporter proteins known as ATP-binding cassettes (ABC) [50,51,52], which potentially limits the cytotoxic effect of chemotherapy. The dysregulation of the ABC protein reduces the uptake or accumulation of chemotherapeutic agents and hinders the drug response in cancer cells, resulting in multi-drug resistance (MDR) [53]. A recent prostate cancer study discovered that the inhibition of ABCC4 of the ABC transporter family improved the sensitivity of docetaxel against resistant cancer cells [54]. Furthermore, ABCB1 expression is significantly downregulated in prostate cancer samples compared to normal prostate. Interestingly, ABCC4 was also upregulated in the prostate cancer samples, suggesting the role of ABCC4 as a primary causative factor of docetaxel resistance [55].

Copper transporter 1 (CTR1) mediates the cellular transport of cisplatin. Abnormal expression of this protein contributes to chemoresistance in cancer [56]. Previous studies have highlighted the robust link between CTR1 expression and cisplatin uptake promoting platinum-based drug (cisplatin, oxaliplatin) resistance in various cancers [57,58,59]. An analysis performed on a Cancer Genome Atlas (TCGA) data set demonstrated an upregulation of CTR1 in response to RNA-binding protein (PTBP1) inhibition, enhancing osteosarcoma cells’ chemosensitivity to cisplatin [60]. Similarly, Wu et al. (2021) demonstrated that zinc-finger protein (ZNF11) down-regulation drastically promoted cisplatin resistance by suppressing CTR1 expression and inhibiting cisplatin influx in epithelial ovarian cancer [61]. Nonetheless, there was no significant correlation between the CTR1 levels and the response to platinum-based drugs due to the broadly similar CTR1 expression in colorectal cancer cells [62]. Thus, the development of CTR1-associated cisplatin resistance is possibly determined by tumour cell type.

One of the major obstacles in modifying aberrant splicing in drug pumps lies in achieving specificity for cancer cells without affecting their normal counterparts. Measures that could help mitigate this challenge are administrating splicing modifying agents (e.g., oligonucleotides) directly into accessible tumours or complexing the oligonucleotides with nanoparticles conjugated to tumour-specific antibodies, thus enhancing targeted delivery.

Detoxification mechanisms: The activation of detoxification systems (metabolic enzymes) can limit chemotherapeutic agent cytotoxicity, protecting cancer cells from environmental toxins [24] and eventually leading to chemoresistance development [63]. One of the primary enzymes responsible for chemotherapeutic agent activation and detoxification is glutathione S-transferase (GSTπ)-catalysed thiol glutathione (GSH) [64]. The GSTπ activation and GSH upregulation are part of the detoxification resistance mechanism, contributing to the onset of chemoresistance by supporting tumour growth and inhibiting the activities of chemotherapeutic agents [65,66,67]. For example, an increase in GSH level in lung cancer cells can inactivate cisplatin by binding to the highly reactive thiol group, preventing the chemotherapeutic agent from binding to DNA and exerting its cytotoxic effect [68]. Furthermore, GSH has several organelle-specific functions as an antioxidant [69]; more than 10% of GSH reacts with reactive oxygen species (ROS) in mitochondria and directly prevents apoptosis [70]. Moreover, an in vitro and in vivo study demonstrated that RNA-mediated depletion of the antioxidant transcription factor Nrf2 hindered ROS detoxification by lowering GSH levels in pancreatic ductal adenocarcinoma, thus enhancing cancer cell sensitivity to gemcitabine [71].

Changes in the drug-induced DNA damage response: Many, if not all, chemotherapeutic agents directly or indirectly damage DNA to activate cell death mechanisms [72]. When DNA damage repair is absent or suboptimal, the DNA repair components are expressed abnormally or become mutated and lead to chemoresistance in various cancers [73]. For instance, poly-(ADP)-ribose polymerase (PARP) is essential to repair DNA-single-strand breakage [74]. A breast cancer study by Hu et al. (2019) demonstrated that the PARP1 inhibitor Olaparib significantly enhanced chemotherapeutic agent (cisplatin and doxorubicin) sensitivity by reducing tumour suppressor BRD7 degradation caused by PARP1 overexpression in resistant cancer cells [75]. Moreover, the abnormal capacity of the nucleotide excision repair (NER) mechanism, a highly conserved DNA repair mechanism, has been proposed as a critical factor in cisplatin resistance across various cancers [76].

Resistant cancer cells often harbour replication protein A (RPA), a key regulator of NER involved in DNA damage [77,78]. Researchers have shown that RPA1 overexpression impeded DNA repair during NER, causing ovarian cancer cells to become cisplatin-resistant. Conversely, silencing RPA1 restored cisplatin sensitivity by promoting Mre11 (double-strand break recognition protein)-dependent degradation of nascent DNA at stalled replication forks [79].

Cell death (apoptosis) evasion: The fundamental cell death mechanism or apoptosis inhibits the survival of damaged or surplus cells [80]. This mechanism is a critical hallmark of chemoresistance [81], as cancer cells evade apoptosis brought about by the administration of chemotherapeutic agents, thus empowering their uncontrollable proliferation [82,83,84]. This escape strategy can be caused by aberrant expression or mutations of apoptotic and anti-apoptotic markers [85] and necrosis, necroptosis and autophagy triggered by chemotherapeutic agents [86]. One of the crucial players orchestrating apoptosis resistance is a group of proteins belonging to the Bcl-2 family. In cancer, the equilibrium between the pro- and anti-apoptotic actions of these proteins is tilted to favour cell survival [87]. A classic example of this disruption has been observed in chronic lymphocytic leukaemia (CLL), where Bcl-2 mutations inhibit apoptosis, ultimately leading to increased survival and chemoresistance in CLL cells [88].

A recent clinical trial found that a Bcl-2 mutation, Gly101Val, markedly reduced the affinity of Venetoclax (Bcl-2 specific inhibitor) to Bcl-2, thus conferring acquired resistance by blocking apoptosis in an estimated 25% of CLL patients. Likewise, Bcl-xL overexpression, another anti-apoptotic protein in the Bcl-2 family, is also responsible for Venetoclax-resistance in the non-Gly101Val mutated cells. Notably, combining Venetoclax with a regimen of chemotherapeutic agents could significantly enhance drug sensitivity in CLL patients by modulating apoptosis regulation and countering acquired resistance [89]. Therefore, targeting Bcl-2 family proteins in clinical trials has been proposed to address cancer resistance, particularly after repeated chemotherapy cycles in CLL and other malignancies.

Extensive studies have demonstrated that the dysregulation of p53, a critical tumour suppressor gene, aids tumour cells in escaping apoptosis upon treatment administration, which reduces DNA damage [90,91,92]. Yao et al. conducted a colorectal cancer study that investigated the role of p53 binding protein 1 (53BP1) loss in driving acquired resistance to 5-Fluorouracil (5-FU). The findings indicated that 53BP1 depletion significantly diminished the 5-FU efficacy by elevating Bcl-2 expression and reducing apoptotic effectors caspase-9 and caspase-3. This mechanism inhibited apoptosis by downregulating the ATM-checkpoint kinase 2-p53 pathway, ultimately contributing to resistance against 5-FU [93].

Despite the increasing understanding of the hallmarks of chemoresistance in devising effective therapeutic responses, the battle to overcome this phenomenon continues as researchers strive to develop safe, sustainable, and effective cancer treatments. The existing literature highlighted that abnormal pre-mRNA AS is potentially critical in shaping the intricate landscape of chemoresistance by significantly regulating gene expression and function involved in resistance mechanisms [94,95]. Therefore, the following subsections explore the complex mechanisms of chemoresistance, particularly the regulatory role of pre-mRNA AS and its involvement in drug resistance development across different cancers. The discussion could shed light on the possible approaches to maximise the effectiveness of chemotherapeutic agents.

## 3. Pre-mRNA Alternative Splicing: A Promising Avenue to Novel Therapeutic Prospects in Cancer Chemoresistance

Approximately 20,000 protein-coding genes in the human genome are responsible for generating over 250,000 protein isoforms, contributing to transcriptomic complexity and proteomic diversity [96]. One of the key mechanisms instrumental in proteomic complexity in eukaryotic cells is pre-mRNA AS. AS is a significant mechanism that processes pre-mRNA through different splicing events, removing introns within pre-mRNA and rearranging exons to produce several mature mRNAs with different structures and functions [97].

**Figure 4:**
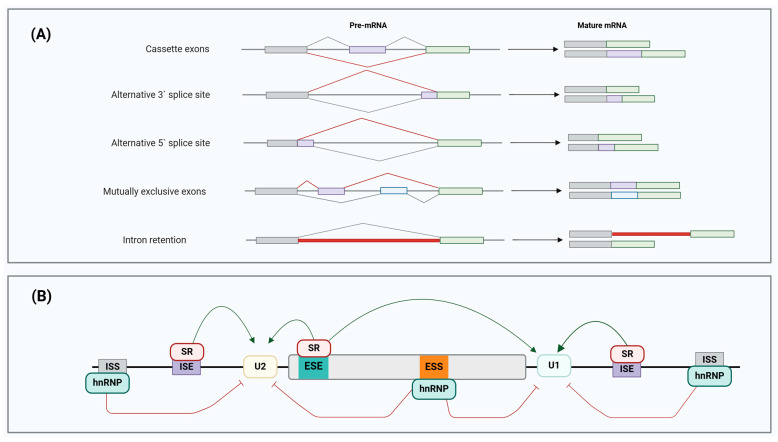
Pre-mRNA splicing regulation. (**A**) Different AS patterns in human tumours. Alternative splicing can be categorised into five primary patterns: cassette exons, alternative 3′ splice site, alternative 5′ splice site, mutually exclusive exons, and intron retention. The exons are represented by the grey, purple, green, and blue boxes, while the different splicing events are demonstrated using grey and red lines. (**B**) Alternative splicing is exhibited as a complex regulatory mechanism driven by the exon-recognition process. RNA splicing is regulated by the complex interactions of cis-acting elements [intronic splice enhancers (ISEs) and silencers (ISSs), exonic splice enhancers (ESEs) and silencers (ESSs)] that recruit RNA-binding protein (RBP) splicing factors and trans-acting proteins [heterogeneous nuclear ribonucleoproteins (hnRNPs) and serine/arginine-rich (SR) proteins] in AS regulation. Adapted from [102]. Created with BioRender.com.

Alternative splicing is classified based on five major patterns of exon configurations found in mature mRNA [98] (see Figure 4A):

- Cassette exons: an exon may be excluded or included;
- Alternative 3′ splice site: a different 3′ splice junction is utilised, changing the 5′ boundary of the downstream exon;
- Alternative 5′ splice site: AS at the 5′ splice site modifies the 3′ splice site of the upstream exon;
- Mutually exclusive exons: different splice variants are generated from various exon combinations, but only one is spliced in the same transcript simultaneously;
- Intron retention: mature mRNA transcript retains the intronic sequence that is present upon the completion of transcript processing, and it becomes a new coding sequence.

All forms of AS are tightly regulated processes that require the ribonucleoprotein (RNP) complex or spliceosome, including cis-acting regulatory sequences and trans-acting regulatory proteins (see Figure 4B) [99]. The spliceosome is a megadalton machinery that catalyses pre-mRNA AS reactions. The formation of the spliceosome involves the intricate assembly of primarily U1, U2, U4, U6, and U5, five primary small ribonucleoprotein particles (snRNPs), which consist of small nuclear RNAs (snRNAs) and their associated proteins, along with over 200 accessory protein factors [100]. Cis-acting regulatory sequences and trans-acting regulatory proteins are vital in recruiting the spliceosome and spliceosomal-associated factors, thereby determining which splice sites are used to establish and maintain the AS patterns [101]. Cis-acting regulatory sequences are classified into four categories based on function and position within the pre-mRNA: exonic splicing enhancers (ESEs), exonic splicing silencers (ESSs), intronic splicing enhancers (ISEs), and intronic splicing silencers (ISSs). Meanwhile, heterogenous nuclear ribonucleoprotein particles (hnRNPs) and serine/arginine-rich (SR) proteins are RNA-binding proteins (RBPs) that act as trans-acting regulatory proteins [102].

The RNA-binding proteins with arginine and serine-rich domains that mediate protein–protein interaction are known as SR proteins [102]. Short splicing occurs when these proteins bind to ESEs and ISEs on pre-mRNA transcripts, promoting the inclusion of particular exons. In contrast, hnRNPs interact with ESSs and ISSs, hence favouring long splicing that inhibits splice site definition and subsequent exon inclusion [103]. Recent reports have described splicing promotion and repression by SR proteins and hnRNPs when binding to different pre-mRNA sites. The selection of a wrong splice site and major alterations in AS patterns can be caused by mutated spliceosomal components, cis-acting regulatory elements, or trans-acting regulatory proteins [104]. Furthermore, the functions of spliceosomal components and RBPs are regulated by kinases and phosphatases through the phosphorylation and dephosphorylation of SR proteins during splice site selection, respectively [105]. In summary, the complex mechanisms of AS are modulated by the delicate interplay between cis-acting regulatory elements and trans-acting regulatory proteins.

## 4. Aberrant Pre-mRNA Alternative Splicing as a Common Hallmark of Cancer

**Figure 5:**
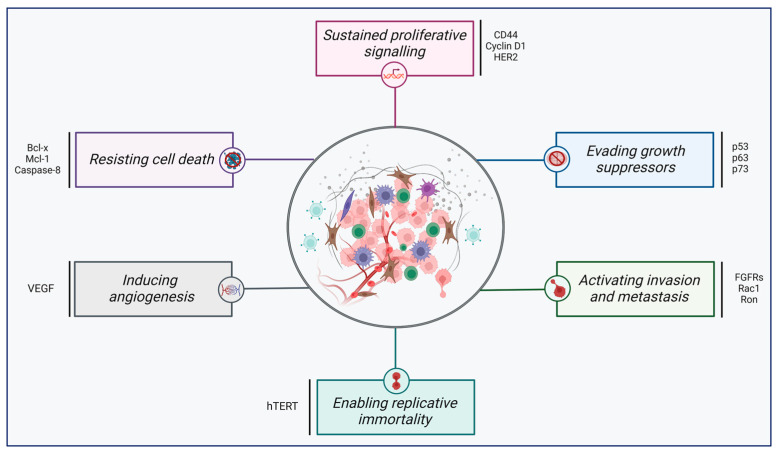
The hallmarks of cancer. This illustration encompasses six hallmark capabilities initially proposed in 2000: (1) sustaining proliferative signalling, (2) evading growth suppressors, (3) activating invasion and metastasis, (4) enabling replicative immortality, (5) inducing angiogenesis, and (6) resisting cell death. Genes impacted by dysregulated alternative splicing are shown next to each hallmark. This figure was adapted from [3]. Created with BioRender.com.

As alterations occur in different hallmarks of cancer and exert oncogenic effects. These cancer hallmarks include sustaining proliferative signalling, evading growth suppressors, activating invasion and metastasis, enabling replicative immortality, inducing angiogenesis, and resisting cell death (see Figure 5) [106,107]. In cancer cells, abnormalities in splicing regulation lead to impaired AS patterns, resulting in aberrant protein and mRNA expression and malignancy progression (Table 2). A deep understanding of the underlying mechanisms involved in abnormal splicing regulation would assist in identifying potential prognostic markers and increase the efficiency of therapeutic strategies to eliminate cancer cells.

The pro-angiogenic factor of the VEGF family, vascular endothelial growth factor (VEGF), is crucial for vascular endothelial cell proliferation and a primary target in cancer treatment. Various splicing sites have been identified in the VEGF-A mRNA. Among the different isoforms, two 3′ AS variant families have been characterised: pro-angiogenic VEGF-Axxxa and anti-angiogenic VEGF-Axxxb isoforms, where xxx represents the number of amino acids encoded [108,109]. The expression ratio of VEGF-A165b/VEGF-A165a was substantially lower in colorectal, breast, kidney, and prostate cancer [110]. Despite the lack of information on the regulatory mechanisms of VEGF-Axxxa and VEGF-Axxxb, overexpression of splicing regulatory factors (SRSF1, SRSF5, SRSF5, and SRPK1) has been associated with the upregulation of VEGF-Axxxa isoforms in head and neck squamous cell carcinomas patients [111]. This finding suggests the AS of regulatory factors can help rectify the differential expression of VEGF pro- and anti-angiogenic isoforms, potentially inhibiting tumour angiogenesis and improving the efficacy of anti-cancer treatments.

A recent study identified compounds that enhanced the expression of the anti-angiogenic VEGF-165b isoform using a splicing-sensitive fluorescent reporter and LOPAC chemical compound library to screen for VEGF-A modulators in cancer cells [112]. These compounds selectively modulate VEGF-A splicing by influencing the inclusion of exon 8, which plays a critical role in the balance between the anti-angiogenic VEGF-A165b and pro-angiogenic VEGF-A165a isoforms. Notably, the study underscored the pivotal role of SRPK in this process, highlighting that the overexpression of SRPK1 favours the pro-angiogenic VEGF-A165a isoform. Conversely, modulating SRPK1 activity with the identified compounds effectively redirected splicing towards VEGF-A165b, thereby inhibiting angiogenesis. Thus, this study not only elucidates the regulatory mechanisms underlying VEGF-A isoform production but also recommends the targeting of VEGF-A splicing as a novel therapeutic approach for cancer and other diseases driven by angiogenesis.

An important target of the epithelial splicing regulatory proteins is the fibroblast growth factor receptor-2 (FGFR-2). This highly conserved transmembrane receptor tyrosine kinase modulates various physiological processes, including angiogenesis, wound healing, cell proliferation, differentiation, and migration. It has been well established that FGFR-2 is alternatively spliced during epithelial–mesenchymal transition (EMT), where epithelial cells acquire mesenchymal characteristics [113]. The FGFR-2 AS pattern is predominantly regulated in a tissue-specific manner. Numerous studies have reported that the abnormal expression of FGFR-2 isoforms induced EMT, prompting a significant increase in malignant phenotypes and tumour invasion and metastasis [114,115]. Similarly, Li et al. utilised a xenograft model of prostate cancer to investigate the impacts of the L-type calcium channel antagonist, nemadipine-A. The study discovered an increased shift from the IIIc isoform to the IIIb isoform. The study also observed a reduction in tumour growth and cell migration [116]. Overall, preventing dysregulation of FGFR-2 AS in cancer may offer novel therapeutic strategies in targeting the EMT.

**Table 2:** Alternative splicing dysregulation in various genes and the impacts on cancer progression.

Gene	Spliced Isoform	Biological	AS Pattern	Cancer	Reference
BIN1	BIN1_12A	Anti-apoptotic	Cassette exons	NSCLC	[109]
CASP2	Casp-2L	Pro-apoptotic	Cassette exons	CRC	[117]
CCND1	Cyclin D1b	Pro-proliferative	Intron retention	HCC	[118]
CD44	CD44s	Pro-invasive	Cassette exons	GBC	[119]
MENA	MenaINV	Pro-invasive	Cassette exons	BRCA	[120]
MKNK2	MNK2-b	Anti-apoptotic	Cassette exons	BRCA	[121]
RAC1	Rac1b	Pro-metastatic	Cassette exons	LUAD	[122]
RON	RONΔ165	Pro-invasive	Cassette exons	CRC	[123]
MNMD4	MDM4-FL	Anti-apoptotic	Cassette exons	BRCA	[124]
FAS	FASL	Pro-apoptotic	Cassette exons	NSCLC	[125]
PKM	PKM2	Promote aerobic glycolysis	Mutually exclusive exons	BRCA	[126]
VEGF	VEGF-165b	Anti-angiogenesis	Alternative 3′ splice site	PC-3	[112]

AS, alternative splicing; NSCLC, non-small cell lung cancer; CRC, colorectal cancer; HCC, hepatocellular carcinoma; GBC, gallbladder cancer; BRCA, breast cancer; LUAD, lung adenocarcinoma; and PC-3, prostate cancer.

Based on the evidence in the existing literature, AS defects can impact various aspects of cancer, including angiogenesis, apoptosis, and metastasis. Nevertheless, studies on patient response to anti-cancer treatment are limited, which has promoted the investigation of cancer cell vulnerabilities associated with AS to enhance treatment efficacy. A better understanding of how abnormal AS influences the participation of splicing factors in regulating gene expression and therapeutic mechanisms could offer valuable insights to develop innovative therapeutic strategies and overcome drug resistance in anti-cancer treatments.

## 5. Unravelling the ‘Rosetta Stone’ of Cancer Chemotherapy: Potential Strategies to Target Abnormal Alternative Splicing

**Figure 6:**
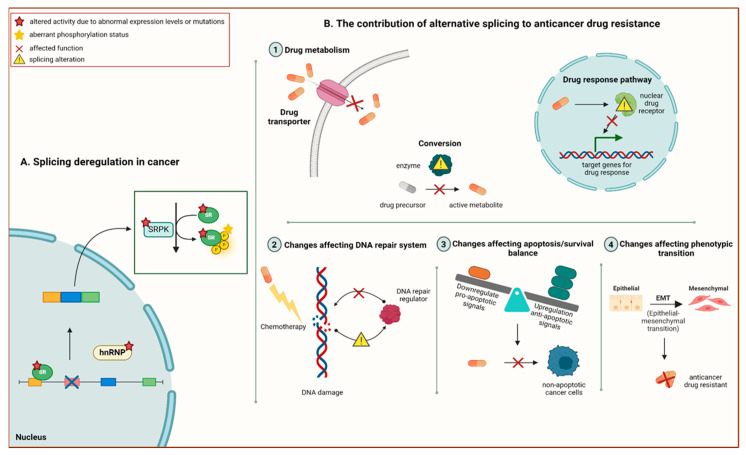
Schematic overview of abnormal alternative splicing contributing to chemotherapy resistance. (**A**) A brief schematic representation of the molecular mechanism resulting in splicing deregulation due to differential expression and mutations of splicing factors in cancer. (**B**) The roles of alternative splicing alterations perturbating anticancer drug response in many aspects. Anticancer drug resistance is shown to be attributed to altered drug metabolism (1), altered DNA damage response (2), altered apoptosis (3), and altered cellular phenotype (4). Adapted from [95]. Created with BioRender.com.

Given the critical role of splicing in cancer progression, splicing errors have become crucial in understanding the poor anti-tumour response to chemotherapy in various cancers. This knowledge has also laid the groundwork for establishing therapeutic strategies to overcome or mitigate chemoresistance and improve patient outcomes [127]. In this section, the latest findings on how AS influences chemotherapy resistance and the potential solutions are divided into four categories based on the disruption in the efficacy of anti-cancer drugs and discussed thoroughly (see Figure 6): (1) AS in altered drug metabolism; (2) AS in alterations of the DNA repair system; (3) AS in alterations of apoptosis/survival balance; and (4) AS in alterations of phenotype transition.

Roles of AS in altered drug metabolism: Understanding the alterations of drug metabolism, including drug targets and transporters and pro-drug conversion into active metabolites, is essential in the ongoing battle against chemoresistance. Recent studies have suggested several alterations derived from abnormal AS within the tumour are the primary cause of resistance to chemotherapeutic agents [128,129]. For instance, the dysregulation of a member of the ABC transporter family, known as ABCG2, has emerged as a prominent factor mediating chemoresistance in various cancers. Moreover, a clinical study involving paediatric patients with acute lymphoblastic leukaemia found that a splicing regulatory variant (G > T) within ABCG2 diminished the transport activity and intracellular concentration of methotrexate (MTX) by disrupting the binding of the SR protein to ESE motifs. Consequently, patient response to MTX was significantly suppressed [129]. A member of the solute carrier transporter family, SLC29A1, also reportedly contributed to chemoresistance. Exon 13 splicing is disrupted by a single-nucleotide alteration in exon 4 of SLC29A1, contributing to the development of cytarabine resistance by inducing reduced cellular accumulation of cytarabine in cytarabine-resistant leukemic cells compared to their matched parental cells in vitro [130].

**Figure 7:**
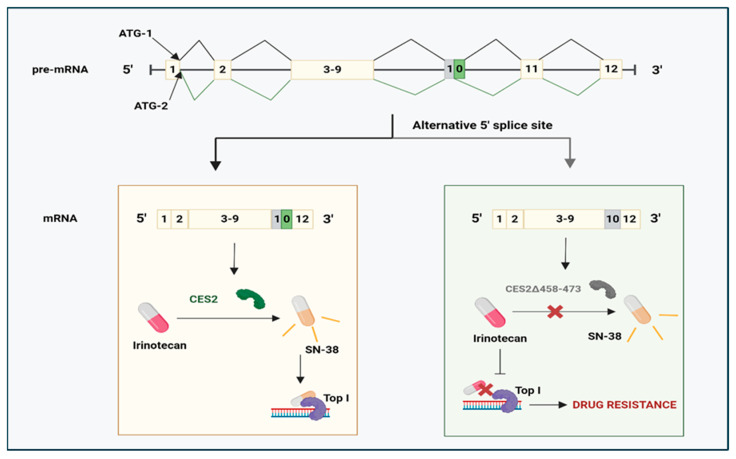
Aberrant CES2 splice variant and its mechanism in chemoresistance. ATG-1: start codon for full-length CES2. ATG-2: start codon for CES2∆ (458–372) splice variant. SN-38: the active metabolite of irinotecan. TOP I: topoisomerase I. Adapted from [134]. Created with BioRender.com.

In addition to drug transporters, abnormal activation of pro-drugs also substantially influences the response to chemotherapeutic agents. The conversion of the pro-drug irinotecan into its active form, SN-38, involves a principle enzyme called carboxylesterase 2 (CES2), which leads to the inhibition of topoisomerase I (TOP I) to induce apoptosis. However, a splice variant within CES2, arising from the lack of 48 nucleotides from exon 10, prevented its conversion into the active form by blocking irinotecan’s hydrolase activity in advanced colon tumour tissue [131]. When the drug cannot bind to TOP I, irinotecan resistance develops (see Figure 7).

Folylpolyglutamate synthetase (FPGS) is a principal enzyme in drug metabolism responsible for converting MTX into MTX-polyglutamate within cells. A study examined whether abnormal AS of the FPGS gene promoted MTX resistance in MTX-resistant acute lymphoblastic leukaemia (ALL) cell lines compared to their parental counterparts [132]. The results revealed a novel variant causing the partial retention of FPGS intron 8, leading to reduced enzyme activity and intracellular MTX-polyglutamylation in MTX-resistant ALL cell lines. Subsequently, a clinical trial found that the overexpression of FPGS’s novel variant was associated with lower accumulation of MTX-polyglutamylation and poorer response to MTX treatment, hence promoting MTX resistance in ALL patient cohorts [133].

**Figure 8:**
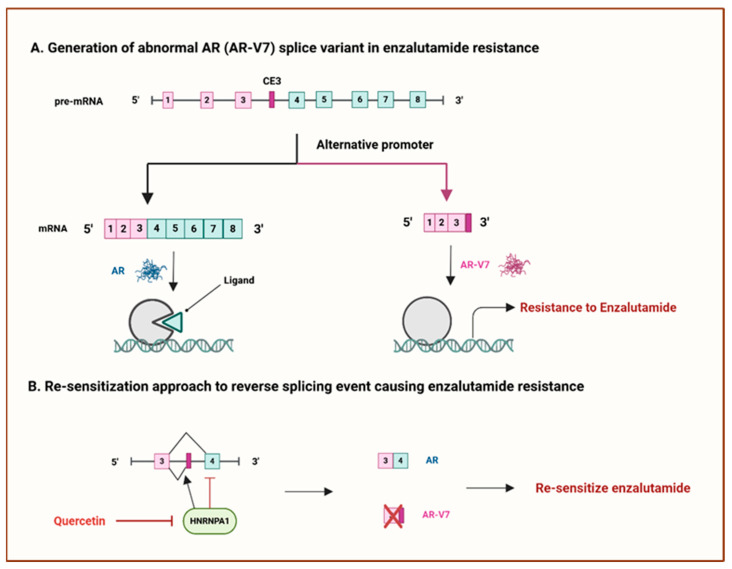
Mechanisms targeting the AR-V7 splice variant in prostate cancer. (**A**) The generation of the AR-V7 splicing variant contributes to enzalutamide resistance. (**B**) Re-sensitisation approach to reverse AR-V7-mediated enzalutamide resistance. CES: cryptic exon 3. AR: androgen receptor. AR-V7: androgen receptor splice variant-7. HNRNPA1: heterogeneous nuclear ribonucleoprotein A1. Adapted from [32]. Created with BioRender.com.

Comprehensive studies are currently being conducted to target abnormal AS in genes encoding drug targets, aimed to eliminate the sources of chemoresistance and enhance treatment response. A classic example of gene alteration resulting from AS is the androgen receptor splice variant (AR-V7), which is the best-characterised androgen receptor (AR) splice variant in advanced prostate cancer. A cryptic exon (CE3) inclusion that brings about a premature stop codon gives rise to this variant. The transcript lacks exons four to eight in the pre-mRNA sequence, forming a functional protein resistant to AR antagonists due to the lack of a ligand-binding domain [135] (see Figure 8A). A recent clinical investigation discovered that the resistance to enzalutamide, an AR antagonist, in metastatic castration-resistant prostate cancer (CRPC) patients was potentially attributed to the AR-V7 splice variant [136]. Since then, an in vitro study has been carried out using enzalutamide-resistant prostate cancer cells to understand and overcome enzalutamide resistance by targeting AS. The study findings indicated that quercetin, a heterogeneous nuclear ribonucleoprotein A1 (hnRNPA1) inhibitor, is highly effective in resensitising enzalutamide-resistant prostate cancer cells to enzalutamide. This compound impedes the AR-V7 splice variant from being generated, which is caused by the abnormal expression of the hnRNPA1 splicing factor [137] (see Figure 8B).

Another significant splice variant linked to chemoresistance is BRAF V600E, a point mutation that occurs in the serine/threonine kinase BRAF and is characterised by the lack of exons 4–8 and the RAS-binding domain (RBD). This RAS-independent modification enhanced the constitutive dimerization of the BRAF V600E isoform, thus causing vemurafenib resistance in melanoma [138]. Additionally, a small molecule inhibitor, spliceostatin A, targets the splicing factor 3B subunit (SF3B1) to inhibit BRAF V600E mutation by competitively binding with the SF3B1 splicing factor. Consequently, the U2 snRNP–SF3B1 spliceosome complex within pre-mRNA could not form in vemurafenib-resistant melanoma cells. This study provided key findings in reversing vemurafenib resistance by regulating AS patterns in melanoma [139,140]. Collectively, these studies illustrated the ongoing efforts to develop effective cancer therapeutic strategies by investigating various alterations in drug metabolism targeting AS to overcome chemoresistance.

Roles of AS in altering the DNA repair: Reduced susceptibility of cancer cells to DNA-damaging chemotherapy is closely linked to aberrantly spliced alterations in the DNA repair system. Breast cancer genes 1 and 2 (BRCA1 and BRCA2) are tumour suppressors involved in homologous recombination repair of DNA-double strand breaks, contributing to chemoresistance development when mutated [141]. Conversely, poly(ADP-ribose) polymerase inhibitors (PARPi) could significantly enhance therapy sensitivity in cancer cells harbouring mutated BRCA1 and BRCA2. In a recent study, the BRCA1-∆11q splice variant, missing most exon 11, which contains an inactivating mutation of the BRCA1 gene, has been attributed to acquired resistance to PARPi, partly diminishing the sensitivity to PARPi and cisplatin in breast cancer. This type of PARPi resistance could be reversed by reducing the levels of the BRCA1-∆11q splice variant via U2 snRNP spliceosome machinery suppression using the small molecule inhibitor P1-B [142]. Selectively targeting this splicing variant is promising in sensitising cancer cells to PARPi treatment. Additionally, the absence of exons 5 and 7 from pre-mRNA caused by exon skipping in BRCA2 gave rise to BRCA2 ∆105+7, an in-frame, novel splice variant with a shorter protein isoform due to the internal deletion of 55 amino acids. This abnormal isoform contributed to mitomycin C resistance in mitomycin-resistant acute myeloid leukaemia cell lines by retaining key functional aspects of BRCA2 [143].

**Figure 9:**
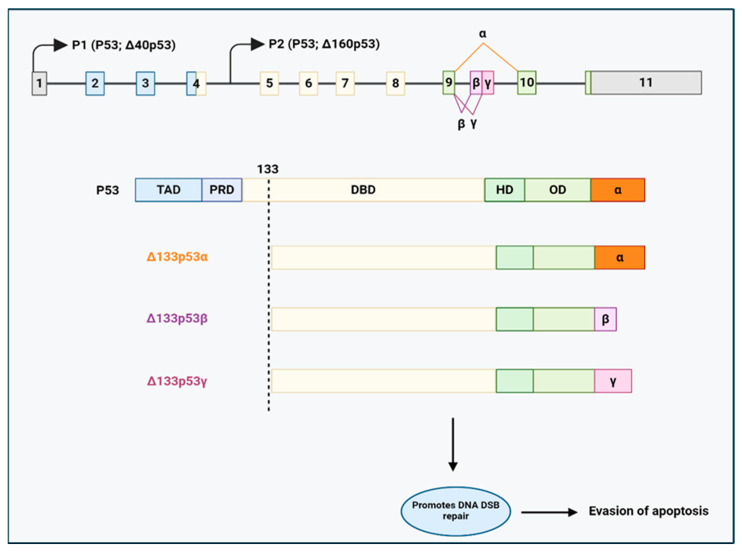
Generation of p53 splicing variants, ∆133p53α, ∆133p53β, and ∆133p53γ, involved in drug resistance of various cancers. P1: promoter 1. P2: promoter 2. TAD: transactivation domain. PRD: proline-rich domain. DBD: DNA binding domain. HD: hinge domain. OD: oligomerization domain. DSB: double-strand breaks. Adapted from [134]. Created with BioRender.com.

Tumour suppressor gene p53 (TP53) is another noteworthy component of the DNA-repair system, and its spliced variants enhance DNA repair and induce chemoresistance in cancer cells. These TP53 splice variants are generated through the AS of TP53, which include p53β (α, β, γ), ∆40p53 (α, β, γ), and ∆133p53 (α, β, γ). The ∆133p53β isoform is generated through an intronic promoter 2 in intron 4, producing the truncated ∆N proteins lacking the initial 132 N-terminal amino acids and undergoing AS at the C-terminus [144,145] (see Figure 9).

A recent study revealed that ∆133p53β was significantly upregulated in glioblastoma tissues, promoting temozolomide resistance and tumour progression [146]. Meanwhile, siRNA-mediated knockdown of ∆133p53 isoform significantly contributed to 5-FU sensitivity in 5-FU resistant cholangiocarcinoma cells [147]. These study outcomes suggest the targeting of abnormal splicing-driven DNA repair systems as an attractive treatment strategy to reverse resistance to chemotherapeutic agents.

Roles of AS in altering apoptosis/survival balance: Apoptosis refers to the cell death programme induced by most of the cytotoxic chemotherapeutic agents. Nevertheless, this mechanism can paradoxically contribute to drug resistance in cancer cells due to an imbalance in pro- and anti-apoptotic components, promoting tumour adaptation when exposed to treatment. Survivin, an apoptosis inhibitor, is vital in the regulation of apoptotic pathways of various cancers. Its overexpression is common in cancer cells, which dampens anti-tumour responses and fuels cancer cell survival and chemoresistance [148]. Alternatively, spliced survivin gives rise to five distinct isoforms, which can be pro-apoptotic (2a and 2b) or anti-apoptotic (∆ex3, 3a, and 3b) [149]. Specifically, the survivin-3B isoform has been associated with the emergence of chemoresistance by escaping the immune response and inhibiting extrinsic and intrinsic apoptotic pathways.

**Figure 10:**
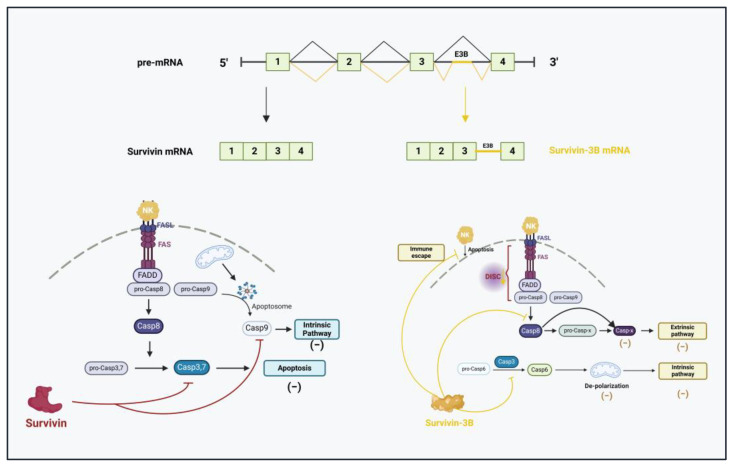
Survivin-3B, a splice isoform of survivin generated through intron retention, enhances the inhibition of the apoptotic pathway by suppressing the pro-apoptotic components caspase-8 and caspase-6, contributing to chemotherapy resistance. NK: natural killer. FASL: FAS ligand. DISC: death-inducing signalling complex. Adapted from [134]. Created with BioRender.com.

Intron retention AS involving 165 bp of intron 3 from pre-mRNA results in the premature termination of translation and the expression of a truncated survivin protein, thus producing survivin-3B [150]. This survivin spliced variant binds to procaspase-8 and inhibits its activation to block the FAS/FASL signalling pathway and prevent death-inducing signalling complex (DISC) assembly. This mechanism promotes cisplatin and 5-FU resistance by suppressing the extrinsic apoptotic pathway in neoplastic cells. In addition, procaspase-3 activity is also affected by survivin-3B, hampering caspase-6 cleavage and disrupting mitochondrial depolarisation. Consequently, the intrinsic apoptosis pathway is blocked, giving rise to cisplatin and 5-FU resistance (see Figure 10) [151,152].

Mcl-1 is another member belonging to the Bcl-2 family that is strictly modulated by AS and important in apoptosis regulation. Aberrant splicing of Mcl-1 generates the anti-apoptotic Mcl-1L (long) instead of the pro-apoptotic Mcl-1S (short), which inhibits apoptosis and promotes cancer progression [16]. A study demonstrated that the overexpression of splicing factors SRSF1, hnRNP K, and hnRNP F/H1 induced splice-switching in Mcl-1, increasing the levels of Mcl-1L in human breast cancer cells. Conversely, the knockdown of these splicing factors increased Mcl-1S expression, thus reducing tumour cell growth and substantially promoting apoptosis [153]. Likewise, in vivo models of gastric cancer exhibited similar outcomes, where dose-dependent treatment of Mcl-1 specific steric-blocking oligonucleotides reduced tumour size and elevated Mcl-1S expression in xenografted mice inoculated with human gastric adenocarcinoma epithelial cells [154]. Therefore, it was proposed that regulating splicing factors involved in AS could promote apoptosis and significantly improve cancer cells’ sensitivity towards chemotherapy.

Pyruvate kinase M (PKM) splicing involves mutually exclusive exons and participates in coordinating the resistance of cancer cells towards chemotherapeutic agents. PKM1 and PKM2 are the two isoforms produced from PKM AS either at exon 9 or exon 10. It is well known that the conversion from the PKM1 isoform to PKM2 isoform promotes cancer cell survival and treatment resistance upon prolonged exposure to chemotherapeutic agents [155]. For example, pancreatic ductal adenocarcinoma (PDAC) cells subjected to prolonged gemcitabine exposure upregulated a critical splicing factor that regulates PKM splicing known as polypyrimidine-tract binding protein (PTBP1), causing PKM2 isoform overexpression. Resultantly, cancer cells acquire the ability to evade apoptosis and enhance their adaptive responses to prolonged gemcitabine exposure. Meanwhile, siRNA-mediated knockdown of PTBP1 sensitised cells to gemcitabine by diminishing its binding to PKM pre-mRNA and reversing the splicing switch towards the PKM1 isoform in gemcitabine-resistant PDAC cells [156]. Thus, targeting differential splicing that favours the PKM2 expression in cancer cells could be useful in preventing chemoresistance by facilitating apoptosis initiation. In summary, these studies highlighted that tilting the balance towards apoptosis promotion can enhance apoptosis in cancer cells, subsequently overcoming chemoresistance.

Roles of AS in alterations of phenotype transition: The EMT is essential in the development of phenotypic plasticity within cancer cells. This plasticity enables cancer cells to gradually develop resistance towards chemotherapeutic agents, posing a major challenge for clinicians to overcome acquired resistance. The best-studied example of EMT involvement in modulating resistance to chemotherapeutic agents through abnormal AS is epithelial splicing regulatory proteins (ESRP1 and ESRP2). ESRP1 suppression induces the formation of the CARM1FL isoform, a full-length transcript of coactivator-associated arginine methyltransferase 1 (CARM1) regulated by ESRP1, giving rise to cisplatin-resistance in small cell lung cancer (SCLC) cells. This spliced isoform led to the inhibition of the TGF-β/Smad pathway through increased phosphorylation of Smad3, thereby promoting EMT in cisplatin-resistant SCLC cell lines [157].

Splicing variants of cluster of differentiation 44 (CD44) are known to accelerate the EMT in cancer cells significantly. A spliced variant of the CD44 gene resulting from exon-skipping, CD44v8-10, participates in chemoresistance development and has been associated with poor prognosis. For example, the upregulation of CD44v8-10 was detected in serum exosomes from CRPC patients. Meanwhile, siRNA-mediated knockdown of CD44v8-10 restored sensitivity to docetaxel, suggesting its potential as a predictive biomarker to monitor resistance against docetaxel in CRPC patients [158]. Octamer-binding protein 4 (OCT4), a key stem cell transcription factor, is often linked to chemoresistance development by driving the EMT in various cancers. The splicing of OCT4 generates the isoform OCT4B1, arising from the inclusion of intronic sequences that contain a premature termination codon. An in vitro study demonstrated that high levels of OCTB4B1 protein expression were significantly linked to increased invasion and metastasis through the upregulating ABCB1 and ABCG2 efflux pumps, ultimately enhancing oxaliplatin resistance in colon cancer cells [159]. These research outcomes indicated that the AS variants exhibit phenotypic plasticity properties, which adds to the complexity of chemoresistance.

## 6. Rationale of Combination Cancer Therapy for Correcting Abnormal Splicing Errors

**Figure 11:**
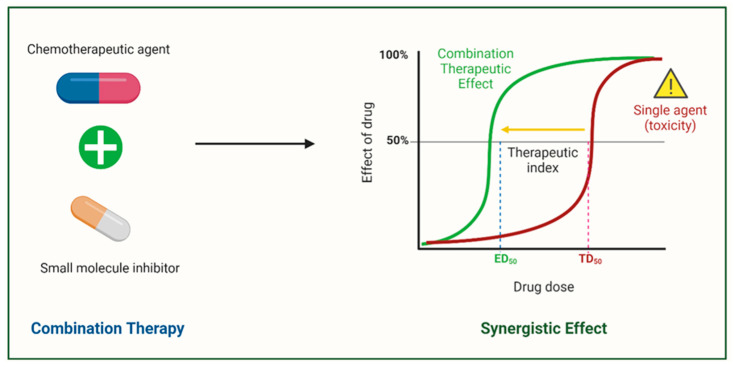
Therapeutic approach that could potentially correct splicing errors. Small molecule inhibitors exert synergistic effects when combined with chemotherapeutic agents, which possibly reduces adverse side effects associated with chemotherapy. Adapted from [95]. Created with BioRender.com. Reversing abnormal AS by targeting splicing factor kinases such as CDC2-like kinases (CLKs) and serine-arginine protein kinases (SRPKs) is one of the most promising strategies to overcome chemoresistance [140]. For instance, SRPIN340, a well-known SRPK inhibitor, blocks SRPK1-mediated hyperphosphorylation of the SRSF1 splicing factor. This inhibition strategy aids in the splicing switch to convert pro-angiogenic VEGF165 to anti-angiogenic VEGF165b in prostate cancer and leukaemia cells [160,161]. Similarly, the SPHINX inhibitor, known as the SRPK1 inhibitor, promoted the conversion of the VEGF165 isoform to the VEGF165b isoform in prostate cancer in an in vivo study, offering an interesting avenue to tackle chemoresistance [162].

The heterogeneity and complexity of chemoresistance mechanisms driven by abnormal splicing errors have prompted the development of combination therapies for cancer patient management. This strategy utilises small molecule inhibitors with unique mechanisms of action in combination with chemotherapeutic agents to overcome resistance mechanisms and improve chemotherapy efficacy (see Figure 11).

Meayamcin B, an SF3B1 inhibitor, also demonstrated the ability to initiate splicing variant switching from Mcl-1L to Mcl-1S through the inhibition of spliceosome assembly. A BH3 mimetic inhibitor of Bcl-xL, ABT-737, was also tested in combination with meayamcin B to treat ABT-737-resistant NSCLC in this study. The combination treatment exhibited a synergistic effect in inducing apoptosis [16]. In conclusion, combination treatment is a promising solution to overcoming chemoresistance and maximising therapeutic efficacy by leveraging the complementary strengths of small molecule inhibitors and chemotherapeutic agents.

## 7. Conclusions

Strategic advancement in cancer patient management continues due to the limitations in clinical response to chemotherapeutic agents. This review provided valuable insights to improve the efficacy of chemotherapy, particularly the modulation of abnormal AS as a therapeutic option in overcoming chemoresistance in various cancers. These observations hold significant relevance, considering these mechanisms are associated with several “hallmark” capabilities of cancer cells. Given the high frequency of aberrant AS linked to chemoresistance, the concomitant application of small molecule inhibitors with chemotherapeutic agents as a cancer treatment is recommended. Future clinical trials should emphasise the understanding of underlying mechanisms linked to aberrant splicing patterns and establish relevant strategies that could improve chemotherapy efficacy and alleviate challenges posed by chemoresistance.

## Figures and Tables

**Table 1:** Limitations of current cancer therapies.

Cancer Therapies	Definition	Limitations	References
Surgery	Removing cancer tissue	Tumour cells can invade distant tissues High risk of recurrence Site dependence Irreversible disfigurement	[16]
Radiation therapy	High-energy radiation is used to eliminate tumour cells	Damages healthy tissues due to the energy placement pattern Resistance-linked side effects	[17]
Targeted therapy	Using pharmacological agents	Difficult to develop drugs against some targets Poor stability for some targets Drug resistance	[18]
Immunotherapy	Targeting tumour- associated antigens to attack cancer cells	Low response rate Cytokine storms Immune-related adverse effects (severe or fatal allergic reactions)	[19]
